# Comparing self- and hetero-metacognition in the absence of verbal communication

**DOI:** 10.1371/journal.pone.0231530

**Published:** 2020-04-28

**Authors:** Laurène Vuillaume, Jean-Rémy Martin, Jérôme Sackur, Axel Cleeremans

**Affiliations:** 1 Consciousness, Cognition & Computation Group (CO3), Universiteé libre de Bruxelles (ULB), Brussels, Belgium; 2 Center for Research in Cognition & Neurosciences (CRCN), Université libre de Bruxelles (ULB), Brussels, Belgium; 3 ULB Neuroscience Institute (UNI), Université libre de Bruxelles (ULB), Brussels, Belgium; 4 École des Hautes Études en Sciences Sociales (EHESS), Paris, France; 5 Subjective Correlates of Cognitive Mechanisms Group (EHESS/CNRS/ENS), PSL Research University, Paris, France; 6 École Polytechnique, Palaiseau, France; Ghent University, BELGIUM

## Abstract

The ability to infer how confident other people are in their decisions is crucial for regulating social interactions. In many cooperative situations, verbal communication enables one to communicate one's confidence and to appraise that of others. However, in many circumstances, people either cannot explicitly communicate their confidence level (e.g., in an emergency situation) or may be intentionally deceitful (e.g., when playing poker). It is currently unclear whether one can read others’ confidence in the absence of verbal communication, and whether one can infer it as accurately as for one’s own confidence. To explore these questions, we used an auditory task in which participants either had to guess the confidence of someone else performing the task or to judge their own confidence, in different conditions (i.e., while performing the task themselves or while watching themselves perform the task on a pre-recorded video). Results demonstrate that people can read the confidence someone else has in their decision as accurately as they evaluate their own uncertainty in their decision. Crucially, we show that hetero-metacognition is a flexible mechanism that relies on different cues according to the context. Our results support the idea that metacognition leverages the same inference mechanisms as those involved in theory of mind.

## 1. Introduction

Metacognition—‘cognition about cognition’—is typically characterized as involving two distinct but interconnected processes: evaluation and control. Metacognitive evaluation involves monitoring the quality of first-order processing, such as memory, perception, language, reasoning and so on [1–10]. Metacognitive control, aimed at improving first-order decisions, can then be deployed based on the outcome of such metacognitive evaluation. For instance, a student who has spent some time studying course materials may judge (metacognitive evaluation) that her mastery of the contents is still insufficient and thus decide (metacognitive control) to continue studying.

Over the last decade, the study of metacognition has essentially focused on the processes and on the mechanisms that underpin intra-personal metacognition (i.e., self-evaluation). However, as is the case for any cognitive information, metacognitive information may be shared with others in order, for instance, to improve collective decision making. For example, when one feels sick, one may lack confidence in one’s ability to self-diagnose accurately and hence decide to go see a doctor to share this uncertainty and have a professional opinion. Recently, different key studies have explored the potential benefit of sharing one’s uncertainty in the context of perceptual decision-making.

Indeed, recent evidence shows that people communicate their metacognitive representations, namely their confidence in their perceptual decisions, and that, under certain conditions, the communication of such metacognitive information leads to improved joint perceptual decisions [11]—this is the “two-heads-better-than-one” effect [5, 11]. Importantly, communication or sharing of confidence is necessary for such joint perceptual decision benefits to occur, even in the presence of external feedback about the accuracy of the perceptual decisions of both subjects of the dyad (conversely, the presence of external feedback is not necessary when confidence is shared) [11]. The beneficial effects of informational exchange between members of a team is not limited to perceptual discrimination and has been shown to improve problem solving [12] or reasoning [13], for instance. Note, however, that in specific conditions the group may not benefit individual performance, as in the *Many Cooks Spoil the Broth* effect. This effect shows that adding more and more expert individuals to a group may eventually undermine both group and individual performance [14, 15].

These results have led some authors to propose that the function of sharing metacognitive representations is precisely to regulate group behaviour [16, 17], a perspective shared by other theoretical proposals [18, 19]: Explicit metacognition makes it possible to regulate interpersonal cognitive control. In addition, in a recent computational account of confidence judgements in one’s first-order performance, Fleming and Daw [20] proposed that intra-personal confidence judgements leverage the very same processes involved when evaluating others’ confidence in their own performance.

So far, research on metacognition and group decision has thus essentially focused on how *communicating* confidence may influence group decision. However, people’s ability to *read* other people’s confidence has so far received little attention. Of course, in many situations, sharing confidence is just a matter of verbal communication: subject A says to subject B how uncertain she is about such or such decision. In many other situations, however, verbal communication cannot be carried out as easily or even trusted. Imagine for instance that you are competing with someone or playing poker. While neither of you *wants* to share information and will in fact deploy efforts to hide information, the ability to read your opponent’s confidence remains nevertheless crucial for your own performance. Similarly, in other daily life settings, such as a romantic date or a job interview, one may not be able to rely as much on verbal communication as on other cues. Likewise, teachers need to be able to carry out online evaluations about whether their students are keeping up with the pace. This potential ability to read others’ uncertainty mental states is in line with substantial research dedicated to the theory of mind ability to read others’ emotions or doxastic states [21]. In this respect, recent work in cognitive neuroscience has found that the neuronal networks that mediate metacognition and mentalizing share common components [22].

In a significant paper, Patel et al. [23] have shown that people are indeed able to read other people’s confidence in a visual discrimination task through the simple observation of the kinematics of other people’s actions, thus, without verbal communication. Participants were shown two intervals that contained six Gabors arranged in a circular fashion around a fixation point. All the Gabors but one had the same contrast, and participants had to decide which interval comprised the “oddball” stimulus. Participants made their decisions by displacing a marble into one of two holes corresponding to the first and the second interval. By means of different sensors, the kinematics of decision-related actions were recorded. The observation task consisted in observing the video-recorded hands of anonymous participants performing the task. Patel et al. [23] have demonstrated that the ability to read others’ confidence from the kinematics of their actions is based on one’s own movement kinematics properties when executing the task oneself. Hence, reading others’ confidence would rest upon motor simulation mechanisms [23].

In the present study, we focused on three main questions: First, are people able to read others’ confidence in the absence of verbal communication and, if this is indeed the case, what are the cues through which this is accomplished? In particular, we surmised that movement cues are not the only cues that people may use when assessing someone else’s confidence, especially when the participant observing the other person also has access to the stimuli. In addition, we hypothesized that observers may also use task difficulty and others’ response time to infer their confidence. Both of these cues have indeed been shown to be important when evaluating one’s own confidence [24, 25] and one may thus expect an observer to use them when evaluating the confidence of someone else. To address this question we developed an ecologically valid paradigm in which people are asked to directly observe actual peers executing the task.

The second question we addressed is whether assessing one’s own confidence is more accurate than assessing someone else’s. In other words, is there a first-person perspective benefit when assessing confidence, or does assessing one’s own confidence leverage exactly the same machinery as that involved when assessing someone else’s confidence ([26]; see also [20])?

The third question we explored is whether inferring the confidence of a participant is more accurate when the observed participant is oneself (by means of a video recording) *versus* someone else. Crucially, stimulus information was not available in this condition, as one could conjecture that the link between task difficulty and confidence is so strong that potential first-person perspective cues are overridden when participants have access to the stimulus.

To address these issues, we designed an auditory pitch discrimination task in which participants had to decide which of two pure tones presented successively had the higher pitch—a first-order decision—and to rate their confidence in their response—a second-order decision. Pairs of participants were tested together. In one condition, participants performed the task separately (Baseline condition); in another condition, while one participant was performing the task, the other was observing her doing it and had access to the stimuli (Full-Observation condition). In what follows the term ‘observer’ denotes the participant observing the other participant performing the task, whom we will call the ‘agent’. In the Full-Observation condition, the observer was to guess the confidence of the agent on each trial (of course, the confidence ratings of the agent were hidden from the observer). In the Partial-Observation condition, the observer was also to guess the confidence of the agent, but she did not have access to the stimuli. Finally, in the Self-Observation condition, each participant observed herself doing the task from a video recording of their Baseline condition, but did not have access to the stimuli themselves. In the three observation conditions, we asked observers to guess the confidence of the agent. Note, however, that due to the lack of stimulus access in some of the conditions, it is not possible to define confidence in a judgement/response. It remains possible that in these cases, observers instead report how uncertain or how fast the agent is instead of how confident they are *per se*. This is in line with recent work distinguishing confidence in a response from general (un)certainty that does not refer to a particular response [27].

We hypothesised that participants would be able to judge the confidence of the agent and that it would be easier for observers to judge agents’ confidence in the Full-Observation condition than in the Partial-Observation condition because of the strong cue that task difficulty constitutes when judging confidence. However, if reading others’ confidence in the absence of verbal communication is indeed possible, the performance of observers should also be predictive of the actual confidence reported by the agent in the Partial-Observation condition. We additionally hypothesised that, in this condition, agents’ response times might constitute a strong cue for observers. As indicated above, response times are an important cue used to infer one’s own confidence [24, 25]. Furthermore, if there is any first-person perspective benefit when assessing confidence, people should be better in evaluating their own confidence in the Baseline condition than when evaluating the confidence of someone else in the Full-Observation condition. Finally, with the same reasoning, people should be better at inferring the confidence of an observed participant when the participant is herself (Self-Observation condition) *versus* someone else (Partial-Observation condition), in the absence of the stimuli.

## 2. Method

### 2.1 Participants

Fifty participants were recruited (Mean Age = 21.3, SD = 1.8). As the experimental task involved pairs of participants, we recruited only female participants so as to avoid gender effects. All participants reported no history of hearing disorder and no history of psychiatric or neurological disorders. Participants received a monetary compensation (10€ per hour). They were naive to the purpose of the study and gave informed consent, in accordance with institutional guidelines. The study was approved by the local ethical committee of the Université libre de Bruxelles.

### 2.2 Apparatus and stimuli

All experimental sounds were sinusoidal pure tones, with 5 ms rise/fall time and 44100 Hz sampling rate, generated using MATLAB (MathWorks, Natick, MA) with the Psychophysics toolbox [28–30]. Auditory stimuli used for the pitch discrimination task were chosen through pilot testing and consisted in a standard pitch sound of 500 Hz which was to be compared to 504, 508, 512, 515 or 518 Hz pitch test sounds. All sounds were played for 250 ms via headphones. The standard stimulus was randomly presented to the left ear or the right ear and the test sound to the opposite ear. The first sound was always presented to the left ear. A fixation cross appeared prior to the sound to signal the beginning of each trial.

### 2.3 Procedure

Participants were paired two by two and did not know each other. Upon arrival, one participant was randomly assigned to first take the role of the agent and the other the role of the observer. They were instructed not to talk to each other.

The experiment was divided in two sessions of approximately 2 hours each. The second session took place between 24h and 48h after the first session. Each session corresponded to two experimental conditions consisting of 250 trials each (with 75 trials for 504 and 508, 50 trials for 512 and 25 trials for 515 and 518 Hz test sounds). In each condition, the agent had to perform the pitch discrimination task and press the left or right arrow on the keyboard with their right hand to indicate which sound had the highest pitch. Participants then had to express how confident they were in their response by pressing a key with their left hand on a separate keypad using a scale ranging from 1 (guess) to 4 (sure). At the end of the session, participants switched roles so that the agent became the observer and the observer became the agent. For each new condition, they returned to their original role assignment.

**Fig 1:**
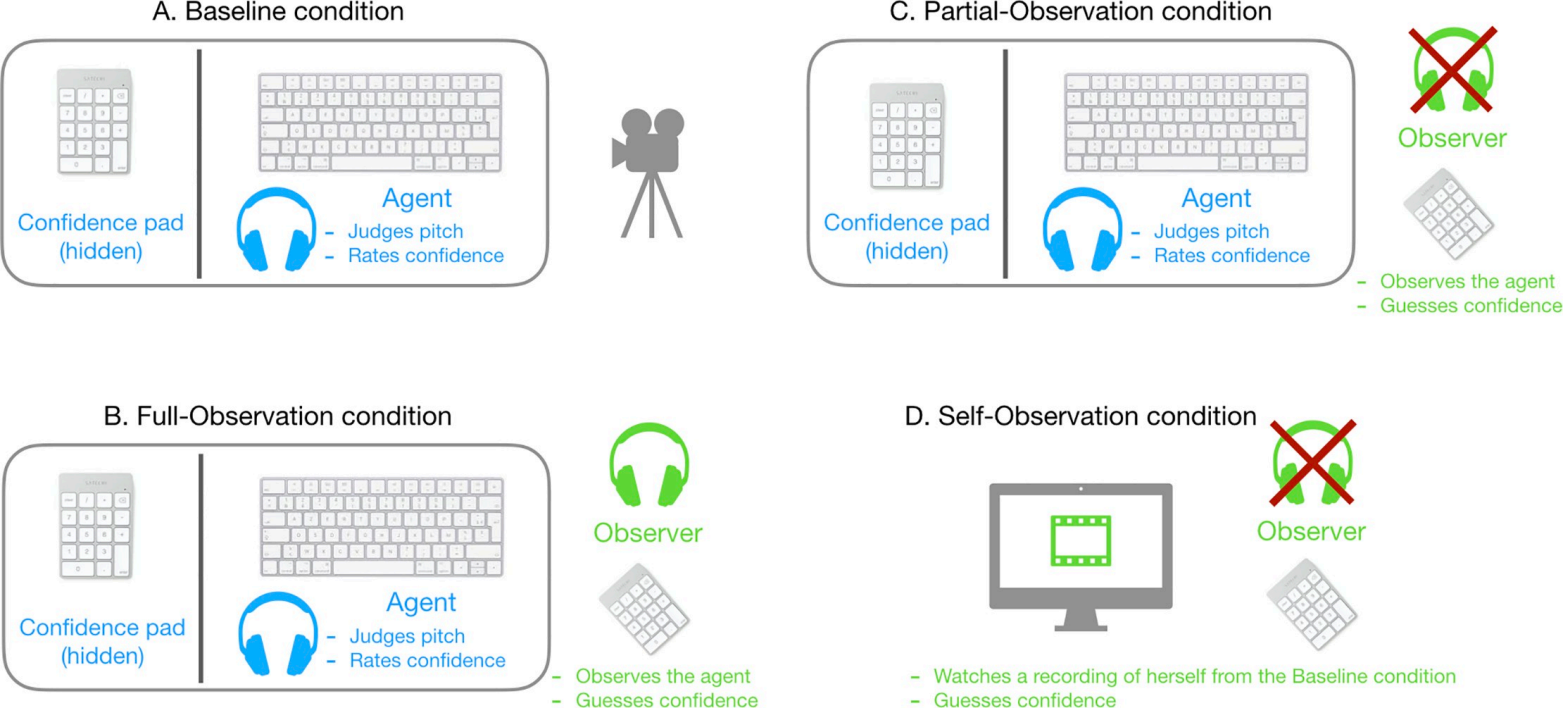
Experimental design. A. Baseline condition: the agent is filmed alone while doing the task; B. Full-Observation condition: the agent does the task while the observer is seated so that she had the same point of view as the camera in the baseline condition. Both the agent and the observer wore headphones and heard the auditory stimuli. Once the agent gave her confidence in her response the observer had to judge what she thought was the confidence of the agent by answering the same confidence scale on her own keypad; C. Partial-Observation condition: the disposition was the same as in the Full-Observation condition except that the observer did not hear the auditory stimuli anymore and wore a sound-proof headset; D. Self-Observation condition: the observer judges the confidence of herself performing the pitch discrimination task in the baseline condition by watching the recorded video without sound.

In the Baseline condition (Fig 1A), participants were seated at a different desk and performed the task on their own without seeing the other participant. During this condition, both were filmed so that their facial expression, body and hands were recorded. In the Full-Observation condition (Fig 1B) participants were seated together at one desk. The observer was seated so that she had the same point of view as the camera in the baseline condition. The keypad through which the agent expressed her confidence ratings was hidden from the observer by means of a cardboard panel. Both the agent and the observer wore headphones and heard the auditory stimuli. Once the agent gave her confidence in her response, the observer had to judge how confident she thought the agent was by using the same confidence scale on her own keypad. The observer could use any strategy that she wanted to guess the agent’s confidence. Once the observer had given her response, a new trial began.

In the Partial-Observation condition (Fig 1C) the disposition was the same as in the Full-Observation condition except that the observer now wore a sound-proof headset which prevented her from hearing the auditory stimuli. However, observers still had access to the response times of the agent, as a fixation cross appeared on the screen to signal the beginning of each trial.

Finally, in the Self-Observation condition (Fig 1D), participants returned to their own desk, as in the Baseline condition. Note that in the Full-Observation, Partial-Observation and Self-Observation conditions, observers could see the agent’s first-order response. Each participant took the role of the observer to judge their own confidence performing the pitch discrimination task in the baseline condition by watching the recorded video without sound. An experimenter was present next to each participant to interrupt the video on each trial (a red cue was presented on the screen once the participant had given her response in the Baseline condition) and restarted the video once a response had been recorded.

The Baseline condition always took place first and the Self-Observation condition always took place last so as to minimize any memory effect in the Self-Observation condition and to ensure that all participants knew the task before judging the confidence of the agent in the ensuing conditions. The order of the Full-Observation condition and the Partial-Observation condition was randomised over pairs of participants. At the end of the third session participants completed the Berkeley expressivity questionnaire [31] in order to assess their emotional expressivity and were then debriefed.

### 2.4 Data preprocessing

Data preprocessing and analyses were performed with R (2016), using the afex [32], lme4 [33], lmerTest [34], BayesFactor [35], ggplot2 [36] and effects [37] packages. One pair of participants was discarded due to issues in the data recording during the experiment. The following analyses were thus made on 48 participants. We also performed additional analyses in Supplementary Material in which we discarded the data from the pairs of participants for which one or both participants had a mean accuracy below 55% or above 95% in at least one condition. To the extent that the present study focuses on metacognition, performances at chance or ceiling might obscure metacognitive analysis. However, these additional analyses actually show that results are almost identical to the analysis performed in the main text on the full sample. We also performed in Supplementary Material preliminary analyses on the influence of the (dis)similarity of performance between participants on the ability to read others’ confidence.

### 2.5 Statistical analysis

In order to compare observers’ ability to assess the confidence of the agent as well as her own confidence in the different conditions, we performed mixed model analyses. We fitted a linear mixed-effects model of the guess of the observer, with confidence of the agent, condition (Full-Observation, Partial-Observation and Self-Observation) and their interaction as fixed and random effect.

In order to test whether the influence of the confidence of the agent on the guess of the observer was mediated by the response times of the agent, we performed mediation analyses. In each condition, a mediator mixed model was first fitted to predict the response times of the agent by the agent’s confidence. Then, an outcome mixed model was fitted to predict the guess of the observer by the response times and the confidence of the agent. The mediation analysis was performed with these two models (using the mediation package; [38]).

Metacognitive sensitivity was estimated through the area under the type-II Receiver Operating Characteristic curve (A_ROC_, [39], for a short overview see [40]). However, here, except in the Baseline condition, we used this measure in a nonconventional way, as we used the guess of the observer and the accuracy of the agent to compute these A_ROC_. This is what we further refer to as the metacognitive sensitivity of the observer regarding the agent.

In addition, we used within-subject repeated measures analysis of variance to test for differences in first-order performances (type 1 sensitivity and criterion, response times) and second-order responses (confidence ratings) and second-order performances (metacognitive sensitivity) followed by paired and one sample t-tests to determine the direction of differences. In all ANOVAs, degrees of freedom were corrected using the Greenhouse-Geisser method.

Finally, we used Bayesian statistics to assess the likelihood that data were in favor of the alternative or null hypothesis using the default medium prior of the BayesFactor R package [35]. This is especially relevant when interpreting non-significant p-values as obtained through conventional statistics [41]. Bayes Factors (BFs) above 3 indicate substantial evidence for the alternative hypothesis whereas BFs below 0.3 indicates substantial evidence for the null hypothesis.

## 3. Results

### 3.1 Agent performance at the first- and second-order level

In order to compare agents’ performance at the first- and second-order level we carried out 6 ANOVAs, as follows (see also S1 Fig).

First, regarding the first-order task (i.e., pitch discrimination task), repeated-measure ANOVAs showed no effect of condition on type 1 sensitivity (*d’*) (Mean = 1.03 and SD = 1.02 in the Full-Observation condition, Mean = 1.06 and SD = 1.02 in the Partial-Observation condition, Mean = 0.88 and SD = 0.77 in the Baseline condition; F(1.13, 53) = 0.61, p > 0.4, η_p_^2^ = 0.01, BF = 0.12) or on criterion (Mean = -0.02 and SD = 0.51 in the Full-Observation condition, Mean = 0.09 and SD = 0.45 in the Partial-Observation condition, Mean = 0.18 and SD = 0.45 in the Baseline condition; F(1.64, 77.18) = 2.45, p = 0.10, η_p_^2^ = 0.05, BF = 0.56). However, a repeated-measures ANOVA showed differences in mean response times across the different conditions (F(1.32, 61.98) = 28.22, p < 10^−3^, η_p_^2^ = 0.38). Specifically, paired *t*-tests indicated that response times were shorter in the Full-Observation (Mean = 1.41 s, SD = 0.33) and Partial-Observation (Mean = 1.37 s, SD = 0.33) conditions than in Baseline condition (Mean = 1.93 s, SD = 0.08) (Full-Observation vs. Baseline: t(47) = 5.37, p < 10^−5^, Partial-Observation vs. Baseline: t(47) = 5.76, p < 10^−6^), but there was no difference between the Partial- and Full-Observation condition (t(47) = -1.02, p > 0.3, BF = 0.26) (note that this decrease in reaction times in the Full-Observation and Partial-Observation conditions compared to the Baseline condition is not associated with an increase in d’, even if the order of the two former conditions is taken into account in the ANOVA (F(1.12,51.64) = 0.12, p > 0.3, η_p_^2^ = 0.003)).

Second, with respect to the second-order task, we found no effect of condition on mean confidence ratings (Mean = 2.83 and SD = 0.46 in the Full-Observation condition, Mean = 2.82 and SD = 0.45 in the Partial-Observation condition, Mean = 2.74 and SD = 0.36 in the Baseline condition; F(1.28, 60.39) = 0.87, p > 0.3, η_p_^2^ = 0.02, BF = 0.14) or confidence ratings variability using standard deviation as a measure of variance (Mean = 0.86 and SD = 0.18 in the Full-Observation condition, Mean = 0.86 and SD = 0.17 in the Partial-Observation condition, Mean = 0.88 and SD = 0.14 in the Baseline condition; F(1.37, 64.55) = 0.44, p > 0.4, η_p_^2^ = 0.03, BF = 0.10). This indicates that the first-order performance and confidence estimates of the agent were not impacted by the different conditions.

Third, we compared the metacognitive sensitivity of the agent across the different conditions using A_ROC_ as a measure of type 2 sensitivity. A repeated-measure ANOVA showed no difference between conditions (Mean = 0.62 and SD = 0.11 in the Full-Observation condition, Mean = 0.62 and SD = 0.11 in the Partial-Observation condition, Mean = 0.60 and SD = 0.09 in the Baseline condition; F(1.29, 60.41) = 1.24, p > 0.3, η_p_^2^ = 0.03, BF = 0.19).

### 3.2 Observer mean confidence level across conditions

In order to compare observers’ confidence level and confidence variability between conditions we performed 2 within-subjects ANOVAs. The mean confidence level of the observer did not differ between conditions (Mean = 2.81 and SD = 0.44 in the Full-Observation condition, Mean = 2.81 and SD = 0.43 in the Partial-Observation condition, Mean = 2.73 and SD = 0.44 in the Self-Observation condition; F(1.67, 78.27) = 1.09, p > 0.3, η_p_^2^ = 0.02, BF = 0.17, S1 Fig) nor did the confidence variability (Mean = 0.83 and SD = 0.17 in the Full-Observation condition, Mean = 0.80 and SD = 0.15 in the Partial-Observation condition, Mean = 0.80 and SD = 0.16 in the Self-Observation condition; F(1.60, 75.04) = 1.13, p > 0.3, η_p_^2^ = 0.02, BF = 0.17).

### 3.3 Observer ability to read agent confidence

In order to compare the relationships between the agent’s and observer’s confidence judgements between the different conditions, we fitted a linear mixed-effects model of the guess of the observer, with confidence of the agent, condition (Full-Observation, Partial-Observation and Self-Observation) and their interaction as fixed and random effects.

The first row in Table 1 (intercept) estimates the average guess of the observer in the Full-Observation condition for the lowest scale rating of the confidence of the agent. The observer had a significantly higher guess about the confidence of the agent than the agent herself when the latter reported guessing (estimate = 2.18, t = 25.65, p < 10^−3^).

**Table 1:** Regression coefficients for the linear mixed-effects model of the guess of the observer in the three conditions.

	Estimate	SE	T	*p*
Intercept	2.09	0.14	14.69	<0.001
Confidence of the agent	0.27	0.03	7.76	<0.001
Partial-Observation condition	0.20	0.13	1.51	0.15
Self-Observation condition	0.13	0.11	1.17	0.26
Confidence of the agent: Partial-Observation condition	-0.09	0.04	-2.28	0.04
Confidence of the agent: Self-Observation condition	-0.08	0.03	-2.46	0.03

Number of participants: 18

Number of observations: 13 500

The second row shows the estimation of the regression slope between the guess of the observer and the confidence of the agent in the Full-Observation condition, and shows that this relation is statistically significant (estimate = 0.21, t = 9.29, p < 10^−3^), indicating that the observer can track the confidence of the agent.

The third and fourth row of the model show that the guess of the observer for the lowest scale rating of the agent was not significantly different between the Self-Observation condition and Full-Observation condition (estimate = 0.12, t = 1.53, p = 0.13), and between the Partial-Observation condition and the Full-Observation condition (estimate = 0.08, t = 0.74, p = 0.46).

Crucially, the fifth and the sixth row indicate that the relationship between the guess of the observer and the confidence of the agent was smaller in the Self-Observation compared to the Full-Observation condition (estimate = - 0.06, t = - 2.67, p = 0.01) and that there was no difference between the Partial-Observation and the Full-Observation condition (estimate = - 0.03, t = - 1.28, p = 0.21).

Another linear mixed-effects model comparing only the Self-Observation condition (in which participants were judging their own performances in the baseline condition by means of video recording) to the Partial-Observation condition revealed no difference in regression slopes (estimate = -0.02, *t* = - 0.90, p > 0.3) between the guess of the observer and the confidence of the agent in the Self-Observation condition compared to the Partial-Observation condition.

In short, those results indicate a decrease in the capacity of the observer to adapt her confidence to the confidence of the agent in the Self-Observation condition compared to the Full-Observation condition. The Partial-Observation condition does not seem to differ from the Full-Observation condition. Finally, there is no difference between the Self- and Partial-Observation condition.

### 3.4 Do observers read agents’ confidence from their response times?

We then asked which cues observers relied on to judge the confidence of the agent. To do so, we explored whether and to what extent the guess of the observer tracked the response times of the agent in the first-order task. The observer could indeed watch the speed with which the agent responded to the first-order task, and use this information to express their confidence.

**Fig 2:**
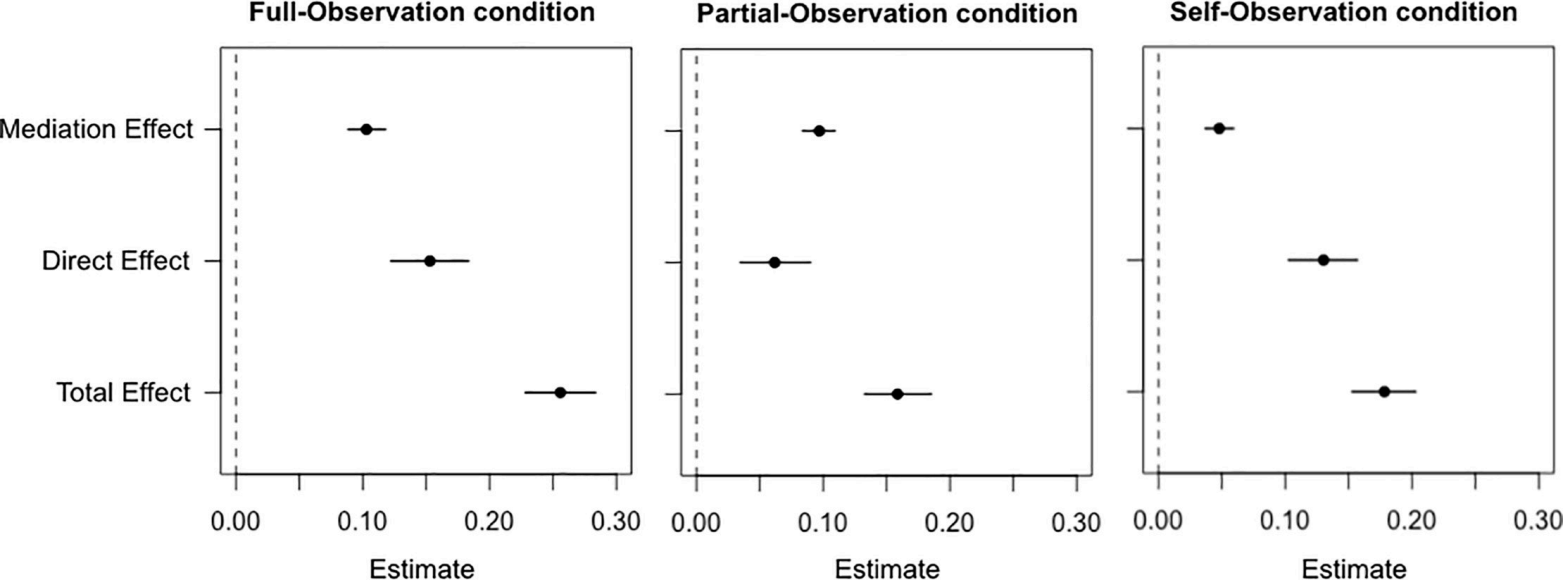
Results of the causal mediation analyses between the mediation and the outcome mixed regression models predicting the influence of the confidence of the agent on the guess of the observer through response times. Error bars reflect quasi-Bayesian 95% confidence intervals.

We used causal mediation analyses to test whether the effect of the confidence of the agent on the guess of the observer was mediated by the response times of the agent (see Statistical analysis; Fig 2). In each condition the mediator mixed model showed a significant relationship between the response times of the agent and the agent’s confidence (Full-Observation: estimate = -0.37, t = -33.1, p < 10^−3^; Partial-Observation: estimate = -0.36, t = -31, p < 10^−3^; Self-Observation: estimate = -0.57, t = -29.7 p < 10^−3^) and the outcome mixed model showed a significant relationship between the guess of the observer and the response times of the agent (Full-Observation: estimate = -0.27, t = -15.6, p < 10^−3^; Partial-Observation: estimate = -0.27, t = -16.2, p < 10^−3^; Self-Observation: estimate = -0.08, t = -8.6, p < 10^−3^) and between the guess of the observer and the confidence of the agent (Full-Observation: estimate = 0.15, t = 10.3, p < 10^−3^; Partial-Observation: estimate = 0.06, t = 4.3, p < 10^−3^; Self-Observation: estimate = 0.13, t = 9.4, p < 10^−3^).

In the Full-Observation condition the mediation analysis showed that from the total effect of the confidence of the agent on the guess of the observer (β = 0.204, 95% CI = [0.186, 0.222], p < .001), there was 42% (95% CI = [38, 45]) that was mediated by the response times of the agent (β = 0.085 95% CI = [0.078, 0.093], p < .001). In the Partial-Observation condition, from the total effect of the confidence of the agent on the guess of the observer (β = 0.172, 95% CI = [0.155, 0.187], p < .001), there was 49% (95% CI = [45, 54]) that was mediated by the response times of the agent (β = 0.084, 95% CI = [0.077, 0.092], p < .001). In the Self-Observation condition, from the total effect of the confidence of the agent on the guess of the observer (β = 0.151, 95% CI = [0.135, 0.168], p < .001), there was 29% (95% CI = [25, 32]) that was mediated by the response times of the agent (β = 0.043, 95% CI = [0.036, 0.049], p < .001).

These findings suggest that the response time of the agent is a crucial mediator between the confidence of the agent and the guess of the observer in the Partial-Observation condition, in which participants did not have access to the stimuli. However, this mediation was partly reduced in the Full-Observation condition, and considerably reduced in the Self-Observation condition. We can draw this inference based on the fact that the 95% confidence interval in the Self-Observation condition does not overlap with the confidence intervals of the other two conditions. In other words, the observer relied less on the response times of the agent to estimate their confidence in the self-observation condition.

### 3.5 Type-II signal detection theory

Thus far, we have shown that participants are able to evaluate other people’s confidence even when they do not have access to the stimuli the agent is judging. In addition, mediation analyses indicated that, when judging other people’s confidence, participants use the agent’s response times, especially when they do not have access to the stimuli. Finally, regression analyses showed a difference between the Partial- and Self-Observation conditions, with a stronger relationship between the confidence of the agent and the guess of the observer in the latter than in the former. This suggests that we do have some kind of privileged access to our own confidence. However, the cues the cognitive system is using differ between conditions, as mediation analyses show that in the Self-Observation condition, response times mediate to a lesser extent the relationship between the confidence of the agent and the guess of the observer. To further corroborate the results of regression analyses between the confidence of the agent and the guess of the observer, we performed type-II signal detection theory analyses (SDT) [42]. Type-II SDT allows to compute the metacognitive sensitivity of individuals, that is their ability to discriminate between their correct and incorrect first-order responses. Here, we reasoned that if participants are able to read others’ confidence, they should be able to discriminate between the correct and incorrect first-order responses of the agent, at least to some extent. We therefore computed A_ROC_ based on the confidence responses given by the observer and the first-order responses of the agent (which corresponds to the same subject in the Baseline condition).

As expected, a one-way one sample t-test showed that the A_ROC_ of participants judging themselves in the Baseline condition were significantly higher than 0.5 (Mean = 0.60, SD = 0.09, t(47) = 7.93, p < 10^−9^). In the Full-, Partial- and Self-Observation conditions the A_ROC_ were also significantly higher than 0.5 (Full-Observation condition: Mean = 0.59, SD = 0.09, t(47) = 7.19, p < 10^−8^; Partial-Observation condition: Mean = 0.53, SD = 0.07, t(47) = 3.58, p < 10^−3^, Self-Observation condition: Mean = 0.53, SD = 0.06, t(47) = 3.01, p < 0.01), suggesting that the metacognitive sensitivity of the observer regarding the agent (or herself through a video recording in the Self-Observation condition) was also higher than chance.

Analysis of variance revealed a significant difference between conditions (F(2.49, 116.90) = 15.25, p < 10^−4^, η_p_^2^ = 0.24), with higher A_ROC_ in the Baseline condition compared to the Partial-Observation condition (paired t-test: t(47) = -4.09, p < 10^−3^) and to the Self-Observation condition (paired t-test: t(47) = -6.60, p < 10^−7^). In the Full-Observation condition we also found higher A_ROC_ compared to the Partial-Observation condition (paired t-test: t(47) = 4.88, p < 10^−4^) and to the Self-Observation condition (paired t-test: t(47) = 4.55, p < 10^−4^) (S1 Fig). However, we found no difference between the Partial-Observation condition compared to the Self-Observation condition (paired t-test: t(47) = 0.57, p > 0.4, BF = 0.18) and no difference between the Baseline condition and the Full-Observation condition (paired t-test: t(47) = -0.42, p > 0.4, BF = 0.17).

In sum, the A_ROC_ analysis largely corroborates the mixed model analysis demonstrating that participants are able to guess others’ confidence in absence of verbal communication. Participants have higher A_ROC_ in the Full-Observation or Baseline condition than in the Partial- or Self-Observation condition (In Supplementary Material, we also propose to compare the meta-d’ between conditions as meta-d’ is now a common measure in metacognition. We found similar results as for the A_ROC_ analysis).

### 3.6 Questionnaire

We found no significant correlations between the emotional expressivity of participants as assessed by the Berkeley expressivity questionnaire and the relationship between the confidence of the agent and the guess of the observer (all ps > 0.5).

## 4. Discussion

In this study, we investigated the extent to which one can evaluate the confidence of others in the absence of verbal communication. We also asked whether one has privileged access to confidence when performing the task directly compared to observing someone else, or when observing oneself compared to observing someone else. We found that people are able to read the confidence of others, even in the absence of verbal communication. We also found that people can guess the confidence of someone else even when they do not have access to the stimuli. Finally, we found that one is not better at self-evaluating one’s own confidence than at evaluating other people’s confidence. Below, we expand on these different results in turn.

First, in line with the study from Patel, Fleming & Kilner [23] (see Introduction), mixed regression analyses showed that in the Full-Observation condition participants (observers) were able to judge the confidence level of agents with a good level of accuracy, indicating that verbal communication as well as fine grained kinematic information [23] are not necessary to share confidence between members of a group. The Type II Signal Detection Theory analysis we carried out in addition to mixed modelling corroborates the fact that participants were able to guess the confidence of someone else with high precision. Indeed, the metacognitive sensitivity (A_ROC_) computed from the guess of the observer and the performance of the agent was significantly above chance in the Full-Observation and Partial-Observation conditions.

Second, participants (observers) were as well fairly good at tracking the confidence level of agents in the Partial-Observation condition in which stimuli were not accessible. The latter finding suggest two, not necessarily exclusive, mind reading processes for confidence: 1) In the Full-Observation condition one could argue that participants are not simply performing the task mentally and inferring the confidence of others based on their own implicit judgements but also base their inferences on the observation of the agent behaviour (see below); 2) One could also suggest that when stimuli are not available (Partial-Observation condition) to participants, they switch to other cues. Mediation analyses suggest that response times had a stronger mediating role in the Partial-Observation than in the Full-Observation condition. However, one has to be careful because confidence intervals of the mediation values overlap between conditions; except with the more conservative dataset in Supplementary Material for which there is no overlap between the Full-Observation and the Partial-Observation conditions. One thus can conjecture that there is a shift in strategy from the Full-Observation condition to the Partial-Observation condition. This phenomenon might be especially important with respect to collective decision, as one may conjecture that response times can thus serve as a competence signal, so that the first person to respond can be the one that will dominate the collective decision.

Note, however, that in the Full-Observation condition, response times are still mediating part of the variance between the actual confidence of the agent and the inferred confidence by the observer. This may reflect two possibilities: First, even in the Full-Observation condition participants do not base their inference of others’ confidence level entirely on their own implicit judgments; second, participants base their inference in relying on their own internal response times explaining the mediating role of agents’ response times and confidence responses of the observers. Future research could further disentangle these possibilities by experimentally manipulating agents’ response times. For instance, one could use confederates playing the role of the agent and purposely responding with incongruent or random response times. This would allow us to see whether the observers still take into account these response times or if they are not disturbed by it under the hypothesis that they are only relying on their own internal response times.

The third important result is that there is no difference in accuracy in assessing confidence level between the Baseline condition, in which participants were performing the task, and the Full-Observation condition in which they were only observing the agent performing the task *plus* having an access to stimuli, as shown by similar metacognitive sensitivity (A_ROC_). Therefore, it seems that, at least in the current experimental design, performing the task oneself does not entail a privileged access in assessing confidence in comparison to observing someone else performing the task. In other words, a first-person perspective does not benefit participants. This could even suggest that one evaluates one’s own confidence like an external observer, that is as when one observes someone else. This finding is in line with current theoretical work [26].

However, it might be that the potential first-person perspective advantage is obscured by the fact that task difficulty constitutes a strong cue in assessing confidence. The access participants (observers) have to stimuli in the Full-Observation condition would equalize confidence accuracy between the latter condition and the Baseline condition. In order to disentangle this point, we compared the Partial-Observation condition to the Self-Observation condition. If there is any advantage at assessing oneself *versus* someone else, we should find that participants are better in the latter than in the former condition. However, both mixed modelling and Type 2 signal detection theory analyses showed no difference between the metacognitive sensitivity of participants in the Self-Observation condition compared to the Partial-observation condition. In addition, using mediation analyses, we found that the mediation effect was the smallest in the Self-Observation condition (in comparison to the Full- and Partial-Observation conditions), with only 29% of the relationship between the confidence of the agent and guesses of the observer mediated by response times. This indicates that when judging themselves, participants used other cues than response times to judge their past confidence. Taken together, these results highlight the fact that metacognitive monitoring (of oneself or someone else) is a flexible process integrating multiple cues and that is responsive to situational demands [25, 43].

Altogether, the present study demonstrates that we can successfully read the confidence of others in the absence of verbal communication and without having access to the information the agent is evaluating. It seems that we can switch between different cues depending on the situation we are in. From an evolutionary perspective, this may be a crucial ability, allowing us to evaluate the confidence of our peers in various situations [16, 17]. A deeper understanding of this phenomenon may also help to shed light on several psychiatric disorders involving difficulties to read others, such as autism [44, 45], schizophrenia [46, 47] or depression [48, 49].

## Supporting information

S1 Fig (TIFF)

S1 File (DOCX)
