# Supplementary figures and images for: Comparing self- and hetero-metacognition in the absence of verbal communication
Source: PLoS One. 2020 Apr 28;15(4):e0231530. doi: 10.1371/journal.pone.0231530 (PMC7188279; doi:10.1371/journal.pone.0231530)

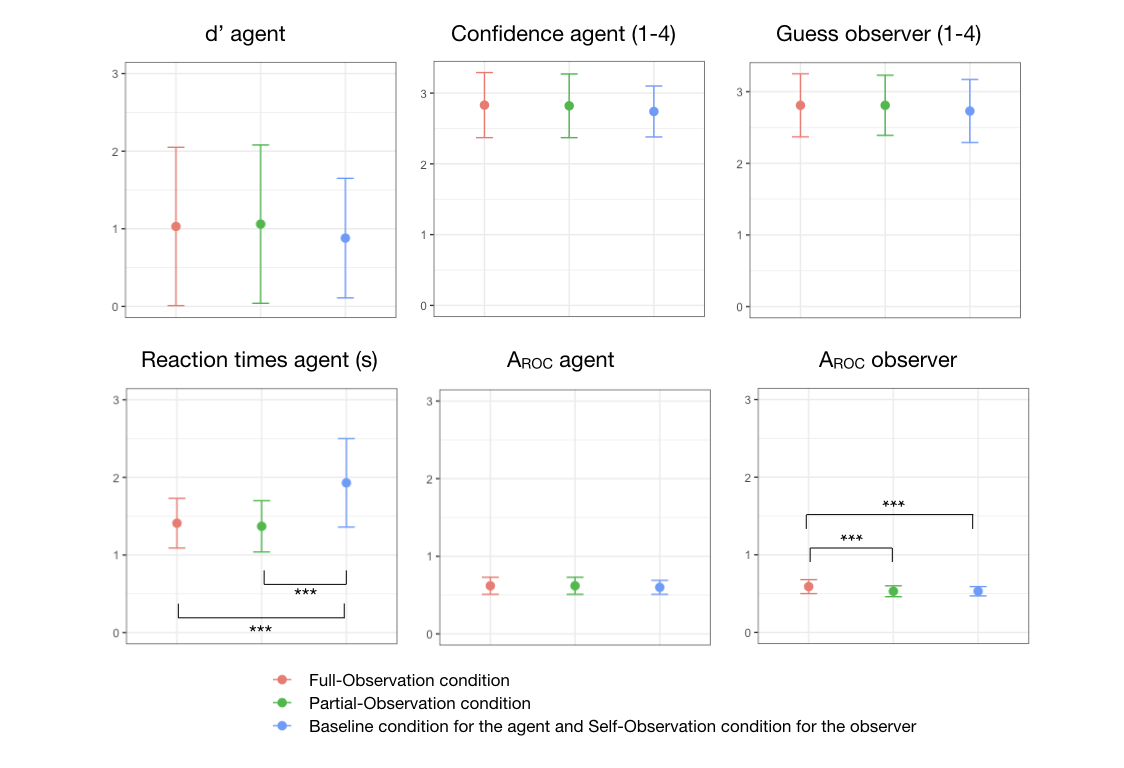

Supplement: S1 Fig — (TIFF) [file pone.0231530.s001.tiff]
