# Supplementary material for: Comparing self- and hetero-metacognition in the absence of verbal communication
Source: PLoS One. 2020 Apr 28;15(4):e0231530. doi: 10.1371/journal.pone.0231530 (PMC7188279; doi:10.1371/journal.pone.0231530)
Supplement: S1 File — (DOCX) [file pone.0231530.s002.docx]

**SUPPLEMENTARY MATERIAL**

**SUPPLEMENTARY ANALYSIS WITH 18 PARTICIPANTS**

As subjects having too high or too low first-order performances might obscure metacognitive sensitivity analysis we conducted additional analyses. They are the same as the main analyses except that we discarded data from pairs of participants for which one or both participants had a mean accuracy below 55% or above 95% in at least one condition. It was indeed not possible to determine individually the level of difficulty as it was critical that participants heard the same stimuli to make their confidence judgements. Twenty-three participants met this exclusion criterion and in total 15 pairs were excluded from analysis. With the pair of participants that was already discarded due to issues in the data recording during the experiment the following analyses were thus made on 18 participants. They reveal that the pattern of results is very similar to results from the main analysis but exhibit stronger effect sizes than the effects described in the main manuscript.

Agent performance at the first- and second-order level

First, regarding the first-order task (i.e., pitch discrimination task), a repeated-measure ANOVA showed no effect of condition on type 1 sensitivity (*d’*) (F(1.10, 18.64) = 1.01, p > 0.3, η_p_² = 0.06) or criterion (F(1.22, 20.79) = 0.35, p > 0.4, η_p_² = 0.02). However, a repeated-measures ANOVA showed differences in mean response times across the different conditions (F(1.39, 23.71) = 14.34, p < 10^-3^, η_p_² = 0.60). Specifically, paired *t*-tests indicated that response times were shorter in the Full-Observation (Mean = 1.41 s, SD = 0.26) and Partial-Observation (Mean = 1.35 s, SD = 0.28) conditions than in Baseline condition (Mean = 1.97 s, SD = 0.53) (Full-Observation vs. Baseline: p < 10^-2^, Partial-Observation vs. Baseline: p < 10^-3^), but there was no difference between the Partial- and Full-Observation condition (p = 0.10, BF = 0.32).

Second, with regards to the second-order task, we found no effect of condition on confidence ratings (F(1.06, 18.05) = 0.65, p > 0.4, η_p_² = 0.04) or confidence ratings variability using standard deviation as a measure of variance (F(1.24, 21.15) = 0.26, p > 0.4, η_p_² = 0.02).

Third, we compared the metacognitive sensitivity of the agent across the different conditions using A_ROC_ as a measure of this type 2 sensitivity. A repeated-measure ANOVA showed no difference between conditions (F(1.51, 25.66) = 1.24, p = 0.30, η_p_² = 0.07).

Observer mean confidence level across conditions

The mean confidence level of the observer did not differ between conditions (F(1.74, 29.64) = 2.80, p = 0.08, η_p_² = 0.14) nor did the confidence variability (F(1.70, 28.85) = 2.70, p = 0.09, η_p_² = 0.14).

Observer ability to read agent confidence

We compared the relations between the agent’s and observer’s confidences between the different conditions. We fitted a linear mixed-effects model of the confidence of the observer, with confidence of the agent, condition (Full-Observation, Partial-Observation and Self-Observation) and their interaction as fixed and random effect.


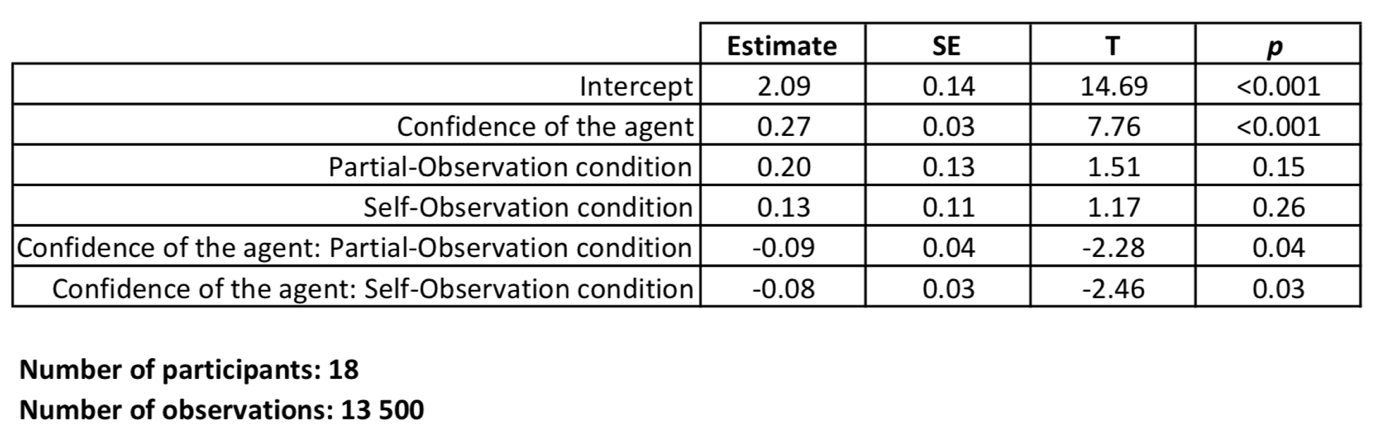


Table S1. Regression coefficients for the linear mixed-effects model of the confidence of the observer in the three conditions.

The first row in **Table S1** (intercept) estimates the average confidence of the observer in the Full-Observation condition for the lowest scale rating of the confidence of the agent. The observer had a significantly higher confidence than the agent when the latter reported guessing (estimate = 2.09, t = 14.69, p < 10^-3^).

The second row estimates the regression slope between the confidence of the observer and the confidence of the agent in the Full-Observation condition, and shows that this relation is statistically significant (estimate = 0.27, t = 7.76, p < 10^-3^), indicating that the observer can track the confidence of the agent.

The third and fourth row of the model show that the confidence of the observer for the lowest scale rating of the agent was not significantly different between the Self-Observation condition and Full-Observation condition (estimate = 0.13, t = 1.17, p > 0.2), and between the Partial-Observation condition and the Full-Observation condition (estimate = 0.20, t = 1.51, p = 0.15).

The fifth and the sixth row indicate that the relation between the confidence of the observer and the confidence of the agent was smaller in the Partial-Observation compared to the Full-Observation condition (estimate = - 0.10, t = - 2.28, p = 0.04) and in the Self-Observation compared to the Full-Observation condition (estimate = - 0.09, t = - 2.46, p = 0.03).

Another linear mixed-effects model comparing only the Self-Observation condition (in which participants were judging their own performances in the baseline condition by means of video recording) to the Partial-Observation condition revealed no difference in regression slopes (estimate = -0.01, t = 0.18, p > 0.3) between the guess of the observer and the confidence of the agent in the Self-Observation condition compared to the Partial-Observation condition.

Do observers read agents’ confidence from their response times

We used causal mediation analyses to test whether the effect of the confidence of the agent on the confidence of the observer was mediated by the response times of the agent. In each condition, a mediator mixed model was first fitted to predict the response times of the agent by the agent’s confidence. Then, an outcome mixed model was fitted to predict the confidence of the observer by the response times and the confidence of the agent. The mediation analysis was performed with these two models (using the mediation package; Tingley, Yamamoto, Hirose, Keele, & Imai, 2014) in order to test whether the influence of the confidence of the agent on the confidence of the observer was mediated by the response times of the agent.

In the Full-Observation condition the mediation analysis showed that from the total effect of the confidence of the agent on the confidence of the observer (β = 0.256, 95% CI = [0.228, 0.283], p < .001), there was 40.1% that was mediated by the response times of the agent (β = 0.103, 95% CI = [0.089, 0.118], p < .001). In the Partial-Observation condition, from the total effect of the confidence of the agent on the confidence of the observer (β = 0.159, 95% CI = [0.133, 0.185], p < .001), there was 61.1% that was mediated by the response times of the agent (β = 0.097, 95% CI = [0.084, 0.109], p < .001). In the Self-Observation condition, from the total effect of the confidence of the agent on the confidence of the observer (β = 0.178, 95% CI = [0.153, 0.203], p < .001), there was 26.9% that was mediated by the response times of the agent (β = 0.048, 95% CI = [0.037, 0.059], p < .001).

Type-II signal detection theory

A one-way one sample t-test showed that the A_ROC_ of participants judging themselves in the Baseline condition were significantly higher than 0.5 (p < 10^-6^). In the Full-, Partial- and Self-Observation conditions the A_ROC_ were also significantly higher than 0.5 (Full-Observation condition: p < 10^-4^; Partial-Observation condition: p < 0.01, Self-Observation condition: p = 0.03), suggesting that the metacognitive ability of the observer regarding the agent (or herself through video recording in the Self-Observation condition) was also higher than chance.

Analysis of variance revealed a significant difference between conditions (F(2.35, 39.99) = 8.08, p < 10^-3^, η_p_² = 0.32), with higher A_ROC_ in the Baseline condition compared to the Partial-Observation condition (paired t-test: p = 0.01) and to the Self-Observation condition (paired t-test: p < 10^-5^). In the Full-Observation condition we also found higher A_ROC_ compared to the Partial-Observation condition (paired t-test: p = 0.03) and to the Self-Observation condition (paired t-test: p = 0.003). However, we found no difference between the Partial-Observation condition compared to the Self-Observation condition (paired t-test: p > 0.4, BF = 0.30) and no difference between the Baseline condition and the Full-Observation condition (paired t-test: p > 0.4, BF = 0.32).

Questionnaire

We found no significant correlations between the emotional expressivity of participants as assessed by the Berkeley expressivity questionnaire and the relationship between the confidence of the agent and the confidence of the observer (all ps > 0.05).

**SUPPLEMENTARY FIGURE**

SUPPLEMENTARY FIGURE

Figure S1. Reaction times, confidence, d’ and A_ROC_ of the agent and guesses and A_ROC_ of the observer between conditions.

**COMPARING META-D’ BETWEEN CONDITIONS**

A repeated measures ANOVA showed differences in meta-d’ between conditions (F(1.93,90.71) = 15.85, p < 10-4, ηp² = 0.25). In the Full-Observation condition we found higher meta-d’ compared to the Partial-Observation condition (paired t-test: t(47) = 5.33, p < 10-5) and to the Self-Observation condition (paired t-test: t(47) = 4.66, p < 10-4). However, we found no difference between the Partial-Observation condition compared to the Self-Observation condition (paired t-test: t(47) = 0.14, p > 0.4, BF = 0.16)

**INFLUENCE OF THE (DIS)SIMILARITY OF PERFORMANCE IN A PAIR**

In order to give preliminary evidence regarding the question of the (dis)similarity between the two participants in a pair with respect to performance level, we additionally took the difference in d-prime between the two participants in a pair and correlated this difference to the slope value between the agent’s confidence and observer’s guess in the Full condition. The correlation is very low and not significant (r = - 0.02, p > 0.7).
